# Numerical Simulation and Process Optimization of Sn-0.3Ag-0.7Cu Alloy Casting

**DOI:** 10.3390/ma19010198

**Published:** 2026-01-05

**Authors:** Hao Zhou, Yingwu Wang, Jianghua He, Chengchen Jin, Ayiqujin, Desheng Lei, Hui Fang, Kai Xiong

**Affiliations:** 1 Materials Genome Institute, School of Materials and Energy, Advanced Computing Center, Yunnan University, Kunming 650091, China; 18406950308@163.com (H.Z.); 18481502377@163.com (A.); lei1632025@163.com (D.L.); xiongkai@ynu.edu.cn (K.X.); 2 School of Engineering, Yunnan University, Kunming 650091, China; 3 Yunnan Tin New Material Company Limited, Kunming 650500, China; hejianghua123@foxmail.com; 4 Kunming Institute of Physics, North Night Vision Science and Technology Research Institute Group Company Limited, Kunming 650223, China

**Keywords:** Sn-0.3Ag-0.7Cu alloy, finite element method, pouring temperature, heat transfer coefficient, porosity

## Abstract

Porosity formation due to solidification shrinkage and inadequate liquid metal feeding during the casting of Sn-0.3Ag-0.7Cu (SAC0307) is a critical issue that impairs quality and subsequent processing. However, the opacity of the casting process often obscures the quantitative relationships between process parameters and defect formation, creating a significant barrier to science-based optimization. To address this, the present study utilizes finite element method (FEM) analysis to systematically investigate the influence of pouring temperature (PCT, 290–390 °C) and interfacial heat transfer coefficient (HTC, 900–5000 W/(m^2^·K)) on this phenomenon. The results reveal that PCT exerts a non-monotonic effect on porosity by modulating the solidification mode, which governs the accumulation of dispersed microporosity. In contrast, HTC plays a critical role in determining porosity morphology by controlling both the solidification rate and mode. Consequently, an optimal processing window was identified at 350 °C PCT and 3000 W/(m^2^·K) HTC, which significantly enhances interdendritic feeding and improves the ingot’s internal soundness. The efficacy of these optimized parameters was experimentally validated through macro- and microstructural characterization. This work not only elucidates the governing mechanisms of solidification quality but also demonstrates the value of numerical simulation for process optimization, offering a reliable scientific basis for the industrial production of high-quality SAC0307 alloys.

## 1. Introduction

Tin-based solder is an indispensable joining material in the modern electronics industry, where its performance directly dictates product reliability and service life [1,2]. Driven by stringent environmental regulations, lead-free solders, such as those in the Sn-Ag-Cu (SAC) system, have become the market mainstream [3,4]. Within this system, SAC0307 has emerged as a compelling choice, offering an excellent balance of properties and economic viability [5,6].

However, realizing the full potential of the SAC0307 alloy is contingent upon the quality of the upstream as-cast ingot, which serves as the starting material for subsequent wire drawing. The casting process is foundational, yet it is where critical defects, particularly porosity, are often introduced [7,8,9]. Beyond solidification shrinkage, metallurgical factors associated with the liquid metal state play a pivotal role in defect formation. Specifically, the solubility of gases in the melt increases significantly with temperature, and excessive oxidation can occur without proper protection. As noted in metallurgical studies, there is a direct correlation between porosity levels and the deoxidation efficacy of the liquid metal; residual gases often precipitate as pores during solidification, exacerbating the defects caused by shrinkage [10]. These internal flaws are directly inherited through downstream processing, compromising the material’s structural integrity and potentially leading to production interruptions.

The fundamental challenge in mitigating these defects lies in the opacity of the casting process itself: critical internal phenomena, such as melt flow dynamics, temperature field evolution, and solidification front propagation, cannot be directly observed. Consequently, process optimization has historically relied on empirical, trial-and-error approaches, which lack the precision needed to systematically establish process-structure-defect relationships. This knowledge gap hinders the consistent production of high-quality ingots [11].

Numerical simulation offers a powerful, science-based framework to bridge this gap by providing high-fidelity insights into the casting process. The utility of this approach is well-documented across a spectrum of applications. For instance, it has been successfully used to optimize traditional casting processes to suppress porosity and misruns in Al-Si-Mg alloys [12]; to explore novel manufacturing methods, such as elucidating the asymmetric solidification characteristics of AA6005 aluminum alloy in twin-roll strip casting [13]; to address the challenges of highly complex scenarios, like the anti-gravity casting of large-scale Ni-based superalloy components [14]; and even to predict location-specific mechanical properties by coupling processing conditions with microstructural evolution, with results validated by experiments [15].

Leveraging these established capabilities, the present study employs the FEM to develop a predictive model for the gravity casting of SAC0307 alloy. The primary objective is to systematically elucidate the influence of two critical process parameters, PCT and the interfacial HTC, on the formation of porosity. By establishing these fundamental relationships, this work aims to define an optimized process window and provide a science-based methodology for improving the internal soundness of industrially relevant SAC0307 ingots.

## 2. Materials and Methods

### 2.1. Numerical Simulation

#### 2.1.1. Physical Process and Governing Equations

In this study, the molten metal during mold filling is treated as an incompressible, Newtonian fluid. This is based on the assumptions that temperature-induced volume changes are negligible and that internal friction (viscosity) due to intermolecular forces is present.

The mold-filling process is characterized by a strong coupling between non-isothermal fluid flow, heat transfer, and localized solidification. Therefore, the governing mathematical model is based on the three fundamental conservation laws: mass, momentum, and energy. The specific governing equations are detailed below [16,17]:

The Mass Conservation Equation states that the mass inflow into a unit volume is equal to the increase in mass within that unit volume. It can be expressed as:

(1) $$\frac{\partial \rho }{\partial t}+\frac{\partial \left(\rho u\right)}{\partial x}+\frac{\partial \left(\rho v\right)}{\partial y}+\frac{\partial \left(\rho w\right)}{\partial z}=0$$

where *ρ* is the density; *t* is time; *u*, *v*, and *w* are the velocity components in the *x*, *y*, and *z* directions, respectively.

The expressions for the Momentum Conservation Equation in a three-dimensional Cartesian coordinate system are:

(2) $$\rho \left(\frac{\partial u}{\partial t}+u\frac{\partial u}{\partial x}+v\frac{\partial u}{\partial y}+w\frac{\partial u}{\partial z}\right)=-\frac{\partial p}{\partial x}+\rho g_{x}+\mu \left(\frac{\partial^{2}u}{\partial x^{2}}+\frac{\partial^{2}u}{\partial y^{2}}+\frac{\partial^{2}u}{\partial z^{2}}\right)$$

(3) $$\rho \left(\frac{\partial v}{\partial t}+u\frac{\partial v}{\partial x}+v\frac{\partial v}{\partial y}+w\frac{\partial v}{\partial z}\right)=-\frac{\partial p}{\partial y}+\rho g_{y}+\mu \left(\frac{\partial^{2}v}{\partial x^{2}}+\frac{\partial^{2}v}{\partial y^{2}}+\frac{\partial^{2}v}{\partial z^{2}}\right)$$

(4) $$\rho \left(\frac{\partial w}{\partial t}+u\frac{\partial w}{\partial x}+v\frac{\partial w}{\partial y}+w\frac{\partial w}{\partial z}\right)=-\frac{\partial p}{\partial z}+\rho g_{z}+\mu \left(\frac{\partial^{2}w}{\partial x^{2}}+\frac{\partial^{2}w}{\partial y^{2}}+\frac{\partial^{2}w}{\partial z^{2}}\right)$$

where *p* is the pressure; *μ* is the dynamic viscosity; *g_x_*, *g_y_* and *g_z_* are the components of the gravitational acceleration.

The Energy Conservation Equation states that the work done by external forces on a unit fluid volume plus the added heat is equal to the total change in energy. The expression is:

(5) $$\frac{\partial T}{\partial t}+u\frac{\partial T}{\partial x}+v\frac{\partial T}{\partial y}+w\frac{\partial T}{\partial z}=\frac{\lambda }{\rho c}\left(\frac{\partial^{2}w}{\partial x^{2}}+\frac{\partial^{2}w}{\partial y^{2}}+\frac{\partial^{2}w}{\partial z^{2}}\right)$$

where *T* is the temperature; *λ* is the thermal conductivity; *c* is the specific heat capacity. The mathematical model established by these equations enables the simulation of a realistic mold-filling process and provides an accurate initial temperature field for the subsequent casting solidification analysis.

#### 2.1.2. Heat Transfer Theory of the Solidification Process

The solidification of a casting involves complex heat transfer phenomena, including convection, conduction, and radiation. Although heat is transferred from the casting to the mold and ultimately dissipated into the environment, thermal conduction is the dominant mechanism governing the casting’s cooling rate and solidification sequence. Consequently, the heat transfer analysis in this study focuses on conduction, which is described by Fourier’s Law of heat conduction [18]:

(6) $$q=-\lambda A\frac{dT}{dx}$$

where *q* is the heat flux (thermal flow rate); *λ* is the thermal conductivity; and (*dT/dx*) is the temperature gradient.

At the interface between the casting and the mold, heat transfer is governed by Newton’s Law of Cooling:

(7) $$q=h(T_{c}-T_{m})$$

where *q* is the heat flux, *h* is the interfacial HTC, *Tc* is the surface temperature of the casting, and *Tm* is the inner surface temperature of the mold.

#### 2.1.3. Mathematical Model of Macroscopic Temperature Field

After mold filling, heat transfer within the casting is dominated by conduction. The solidification process is therefore governed by the transient, three-dimensional heat conduction equation [19,20]:

(8) $$\frac{\partial }{\partial x}\left(\lambda \frac{\partial T}{\partial x}\right)+\frac{\partial }{\partial y}\left(\lambda \frac{\partial T}{\partial y}\right)+\frac{\partial }{\partial z}\left(\lambda \frac{\partial T}{\partial z}\right)+\rho L\frac{\partial f_{s}}{\partial t}=\rho c\frac{\partial T}{\partial t}$$

where *λ* is the thermal conductivity; *T* is the temperature; *ρ* is the density; *c* is the specific heat capacity; t is the time; *Q* is the internal heat source term.

In solidification analysis, the internal heat source term (*Q*) represents the release of latent heat. Thus, Equation (7) can be rewritten to explicitly include the latent heat of solidification (*L*) and the evolution of the solid fraction (*fₛ*):

(9) $$\frac{\partial }{\partial x}\left(\lambda \frac{\partial T}{\partial x}\right)+\frac{\partial }{\partial y}\left(\lambda \frac{\partial T}{\partial y}\right)+\frac{\partial }{\partial z}\left(\lambda \frac{\partial T}{\partial z}\right)+\rho L\frac{\partial f_{s}}{\partial t}=\rho c\frac{\partial T}{\partial t}$$

where L is the latent heat of solidification; f_s_ is the solid fraction.

#### 2.1.4. Porosity and Shrinkage Criteria

The formation of porosity and micro-shrinkage defects during the casting process is primarily due to an insufficient liquid metal feeding during the final stages of solidification. In the finite element software, the Niyama criterion is employed for the prediction of these defects [21,22]:

(10) $$\frac{G}{\sqrt{R}}<C_{Ny}$$

where *Ny* is the Niyama criterion value; *G* is the local temperature gradient, calculated as

(11) $$G=\max\left(\frac{T_{i,j,k}-T_{i+m_{i},j+m_{j},k+m_{k}}}{{\left(m_{i}+\Delta x\right)}^{2}+{\left(m_{j}+\Delta y\right)}^{2}+{\left(m_{k}+\Delta z\right)}^{2}}\right)$$

*R* is the cooling rate, calculated as

(12) $$R=\frac{T_{i,j,k}^{m}-T_{i,j,k}^{-m}}{\Delta t}$$

*C_Ny_* is the critical value for porosity formation, in the range of 1.0 to 1.1.

#### 2.1.5. Simulation Setup

**Figure 1:**
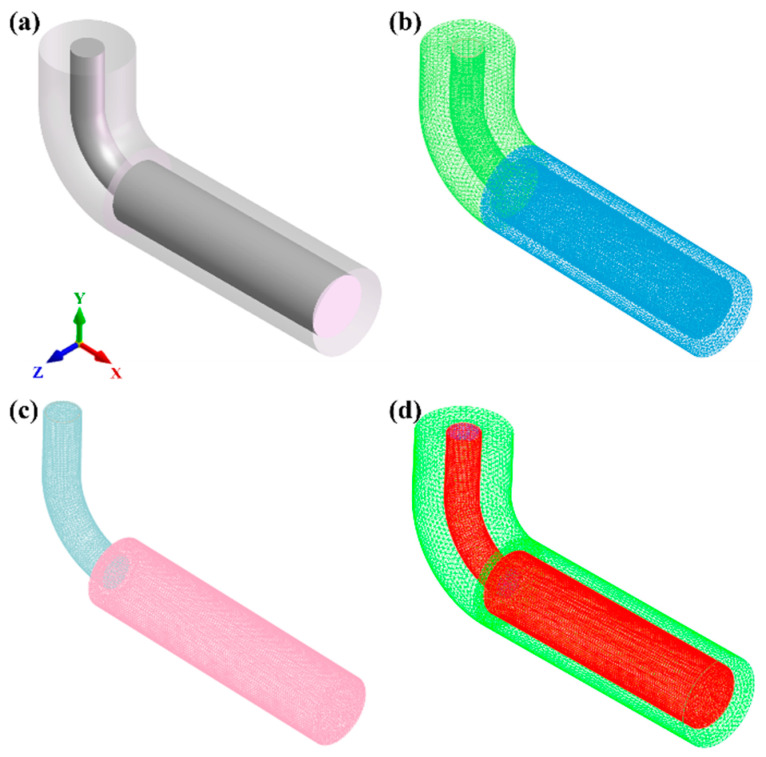
Three-dimensional (3D) model and volume mesh discretization of the casting system: (**a**) geometric setup, (**b**) mold volume mesh, (**c**) casting volume mesh, (**d**) heat transfer interfaces.

The three-dimensional (3D) geometry of the casting model is shown in Figure 1a. The model consists of a cylindrical ingot (radius *r* = 49 mm, length *L* = 400 mm) and an attached pouring runner. A complete 3D model of the mold and casting assembly was constructed using specialized software and exported in Initial Graphics Exchange Specification (IGS) format. Subsequently, this file was imported into the meshing module of the finite element software for simulation. The meshing procedure involved generating the surface mesh from the imported model, checking its quality, and finally generating and verifying the final volume mesh.

To balance computational accuracy and efficiency, a graded meshing strategy was used. The mesh size for the mold was set to 8 mm, while a finer mesh size of 4 mm was applied to the ingot (casting body). The final discretized mesh model, as presented in Figure 1b,c, comprised a total of 36,216 surface elements and 407,982 volume elements. Furthermore, the heat transfer interfaces defined between the casting and the mold, which are critical for the thermal coupling calculation, are illustrated in Figure 1d.

**Figure 2:**
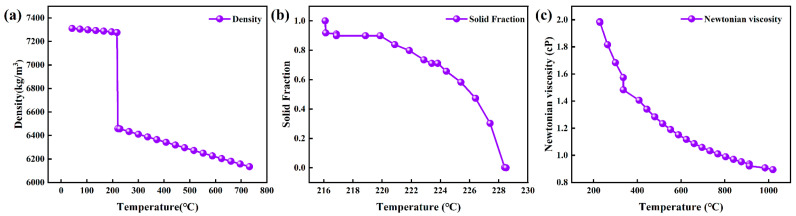
Temperature-dependent curves of thermophysical data of SAC0307 alloy: (**a**) Density; (**b**) Solid fraction; (**c**) Newtonian viscosity.

The material investigated is the low-silver solder alloy, SAC0307. Its chemical composition is detailed in Table 1, was experimentally determined using Inductively Coupled Plasma Optical Emission Spectroscopy (ICP-OES). The alloy exhibits a solidus temperature of 217 °C and a liquidus temperature of 228 °C. The temperature-dependent thermophysical properties of the alloy, including density, solid fraction, and Newtonian viscosity, are shown in Figure 2. The simulation models the gravity casting process where the molten alloy is poured to obtain an ingot, with a target mass for a single cast blank of approximately 22.573 kg.

The mold material is low-carbon steel (AISI 1008), chosen for its favorable thermal conductivity and excellent resistance to hot cracking. Crucially, the horizontal end of the mold was equipped with a water-cooling system. This configuration effectively functions as a chill mold, specifically designed to induce non-uniform heat transfer and establish a steep longitudinal temperature gradient. This gradient enforces directional solidification, which is essential for facilitating interdendritic feeding to compensate for solidification shrinkage and for promoting the development of the characteristic dendritic microstructure. The simulation was configured to reflect these physical conditions with the following key boundary conditions and process parameters. Gravity was applied in the negative Y-direction. The initial PCT was set to 330 °C, while the initial temperatures for the top and horizontal ends of the mold were 178 °C and 43 °C, respectively. A constant Interfacial HTC of 2000 W/(m^2^·K) was defined between the casting and the mold to capture the rapid heat extraction. This value and the investigated range (900–5000 W/(m^2^·K)) align with typical HTC values reported in the literature for gravity casting in permanent molds, which can reach up to 3000–4000 W/(m^2^·K) at water-cooled interfaces depending on contact conditions [23,24]. For the thermal boundary conditions, the top end of the mold was treated as adiabatic, whereas the horizontal end was subjected to water cooling. The mold filling process was specified to complete within 15 s. Finally, the simulation was set to terminate either when the entire casting cooled below 206 °C or after reaching a maximum of 50,000 calculation steps.

### 2.2. Experiments

Cylindrical ingots of SAC0307 alloy (composition detailed in Table 1) were produced by gravity casting in a water-cooled permanent steel mold. As-received SAC0307 ingots were melted at 330 °C in a resistance furnace, stirred, Crucially, to prevent the entrapment of oxide inclusions and ensure ingot integrity, the surface dross was carefully skimmed immediately prior to pouring. The melt was then poured through a runner into the mold over 15 s, yielding ingots weighing approximately 22.573 kg. The mold, made of AISI 1008 steel, was water-cooled at the horizontal end to act as a chill, thereby establishing a steep temperature gradient to promote directional solidification. After cooling to room temperature, longitudinal sections were extracted via Wire Electrical Discharge Machining (WEDM). These sections underwent standard metallographic preparation, involving grinding followed by polishing with diamond and finally silica suspensions. The prepared surfaces were subsequently examined using a Zeiss Axio Vert.A1 inverted optical microscope (OM) to characterize the dendritic microstructure.

## 3. Results and Discussion

### 3.1. Temperature and Solid Fraction Evolution During Solidification

**Figure 3:**
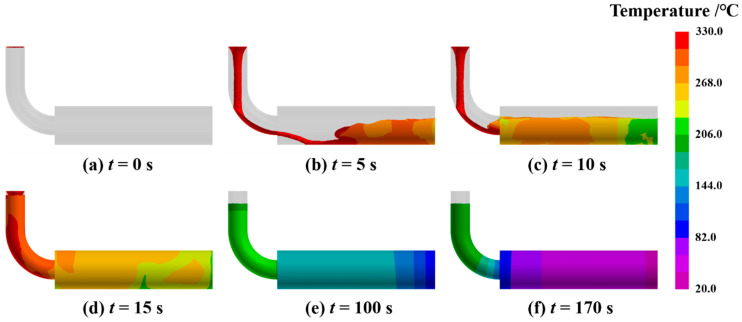
Simulation results showing the temperature field evolution during the mold filling and solidification process, (**a**) 0 s, (**b**) 5 s, (**c**) 10 s, (**d**) 15 s, (**e**) 100 s, (**f**) 170 s.

The simulated evolution of the temperature field during the mold filling and solidification process is presented in Figure 3. Immediately after pouring, the molten alloy flows through the runner into the horizontal mold cavity. Due to the high pouring velocity and the relatively small diameter of the runner, turbulent flow is initially generated within the cavity. As filling progresses, the melt level rises steadily. Once filling is complete, the molten metal begins to cool and solidify. During the initial stage of solidification, the large temperature differential between the melt inside the cavity and the mold material drives a rapid rate of heat conduction. As the ingot gradually solidifies, this temperature difference decreases, causing the cooling rate to slow down. This simulated thermal profile, characterized by a rapid initial temperature drop followed by a solidification plateau, shows excellent agreement with classical cooling curves reported for gravity casting of similar alloys [25]. This consistency validates the accuracy of the thermophysical parameters and thermal boundary conditions applied in the simulation, confirming that the model effectively captures the actual heat dissipation dynamics. The simulation concluded after approximately 170 s, at which point the entire casting had solidified and cooled to the termination temperature of 206 °C.

**Figure 4:**
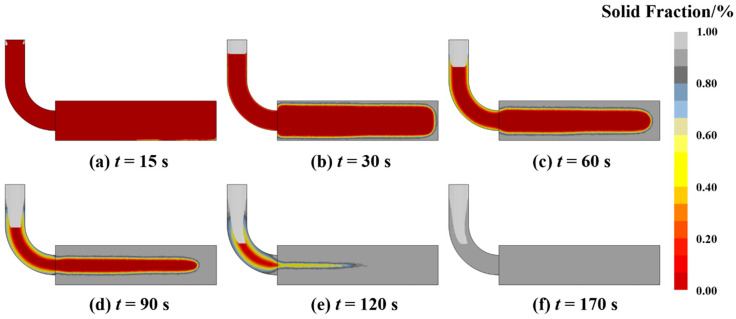
Simulation results of solid fraction evolution during the solidification process, (**a**) 15 s, (**b**) 30s, (**c**) 60 s, (**d**) 90 s, (**e**) 120 s, (**f**) 170 s.

The evolution of the solid fraction throughout the casting solidification process is presented in Figure 4. Solidification initiates at the ingot edges due to direct thermal contact with the mold, after which the solidification front progresses inward from the surface toward the central axis. As illustrated in Figure 4d,e, as the process advances to 90 s and 120 s, the unsolidified melt in the core evolves into a narrow, elongated liquid channel. This morphology is characteristic of a deep U-shaped solidification front, a geometry recognized in classical casting theory as a critical precursor to centerline porosity. Existing studies have indicated that such restricted residual liquid regions are prone to “solid bridging” between adjacent dendrite arms, particularly during the late stages of solidification [26], such restricted residual liquid regions are highly prone to “solid bridging” between adjacent dendrite arms, particularly during the late stages of solidification. Unlike a planar front that facilitates efficient feeding, this deep U-shaped profile extends the mushy zone, creating a scenario where dendritic bridging effectively segments the liquid channel. This segmentation drastically increases the hydraulic resistance to feeding flow, creating isolated pockets where volumetric shrinkage cannot be compensated by liquid inflow. While the macroscopic simulation confirms that no large isolated liquid pools were initially formed, the identification of this specific solidification mode provides a robust physical basis for linking the simulated thermal field to the macroscopic shrinkage defects observed in experiments.

### 3.2. Porosity Prediction and Analysis

**Figure 5:**
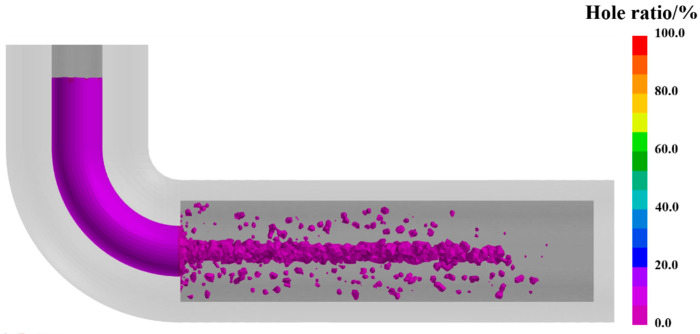
Simulated distribution of shrinkage porosity within the cast ingot.

The simulated distribution of porosity defects is displayed in Figure 5. As shown, the defects are predominantly concentrated along the central axis of the ingot, manifesting as a large, continuous band of macro-shrinkage, while peripheral regions remain relatively sound with only minor, dispersed microporosity observed at the far right end. This distinct distribution pattern is intrinsically linked to the solidification dynamics governed by the water-cooling boundary condition, where the directional solidification front progresses from the outer surface toward the thermal center. Consistent with the mushy zone evolution described in previous studies [27], the simulation captures the formation of a deep, narrow U-shaped mushy zone along the central axis during the final solidification stage. Within this constricted region, the flow of residual liquid metal required to feed solidification shrinkage is severely impeded. As explained by the momentum transport theory for interdendritic flow [28], the hydraulic resistance in such a dense dendritic network is dominated by the Darcy term, leading to a substantial pressure drop. When the local pressure falls below the cavitation threshold or when the feeding path is completely cut off by solid bridging, the volume deficit created by the thermal contraction of the metal cannot be compensated, resulting in the observed centerline porosity. The agreement between this theoretical mechanism and the simulated defect morphology confirms that the model parameters adequately represent the physical solidification conditions. These findings highlight that the inherent solidification path under initial conditions is insufficient to ensure internal soundness. Therefore, to eliminate these porosity defects, it is necessary to optimize the process parameters, specifically the PCT and the HTC. By adjusting the solidification mode, the occurrence of defects can be prevented.

### 3.3. Effect of Pouring Temperature on Porosity

**Figure 6:**
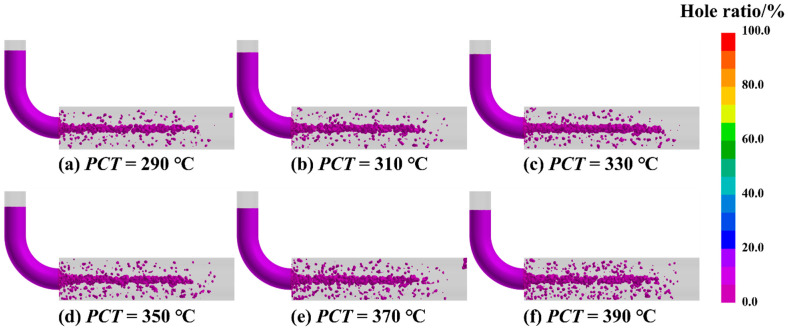
Simulated distribution of shrinkage porosity at different PCTs, (**a**) 290 °C, (**b**) 310 °C, (**c**) 330 °C, (**d**) 350 °C, (**e**) 370 °C, (**f**) 390 °C.

**Figure 7:**
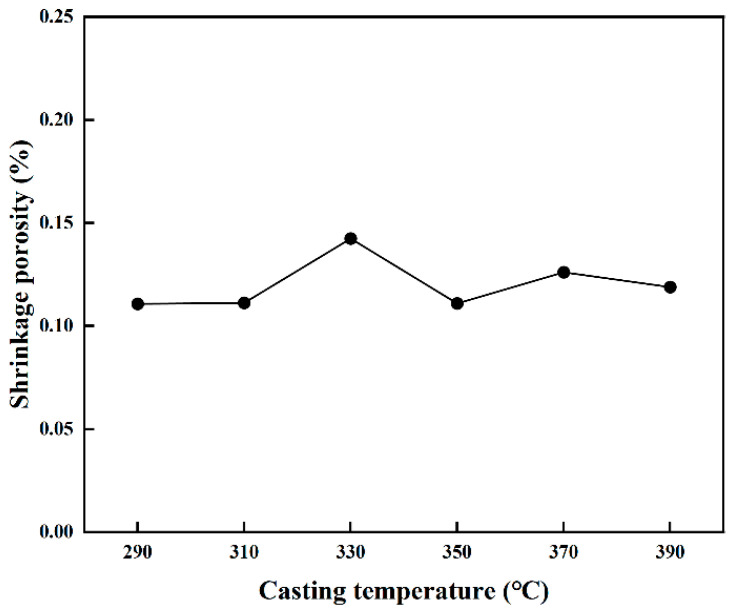
Porosity fraction as a function of PCT.

PCT acts as a decisive process variable that fundamentally determines the mold-filling capability and the susceptibility to defect formation during casting solidification. The influence of PCT on the internal soundness of SAC0307 alloy ingots was examined across a range of 290 °C to 390 °C, adhering to the principle that PCT should be maintained 50 °C to 150 °C above the liquidus temperature. As depicted in the simulated distribution and statistical results shown in Figure 6 and Figure 7, the total porosity fraction follows a non-linear trajectory, rising to a distinct maximum at 330 °C before subsiding to a minimum at 350 °C, followed by a slight resurgence at higher temperatures. This complex variation is governed by the competitive interplay between hydraulic feeding efficiency and gas solubility, a dual-mechanism phenomenon consistent with recent findings on porosity formation [29]. Specifically, at lower temperatures approaching 330 °C, the melt exhibits higher viscosity and rapid cooling, which accelerates the growth of the dendritic network and promotes premature solid bridging. Consistent with the channel blockage mechanism described in solidification theory, this structural impedance effectively isolates pockets of residual liquid from the feeding path, thereby exacerbating shrinkage porosity. Conversely, the reduction in defect volume at 350 °C is attributed to improved fluidity, where higher superheat delays dendrite coherency and prolongs the duration of effective liquid feeding. This aligns with previous research demonstrating that finer grain structures at channel tips, often associated with optimized thermal conditions, significantly enhance flow length and fluidity. However, further increasing the temperature beyond this optimum reintroduces defects. This observation aligns with Sievert’s law, which states that the solubility of gases in the melt increases with temperature. The elevated superheat exponentially enhances the solubility of gases, leading to microporosity precipitation upon solidification [30]. Additionally, as noted in studies on melt quality [10], excessive superheat can promote the formation of oxide inclusions. These inclusions not only degrade fluidity but also act as nucleation sites for gas bubbles, further contributing to the observed resurgence in porosity. Consequently, 350 °C is determined to be the optimal processing parameter, effectively balancing the reduction in shrinkage porosity via enhanced feeding against the mitigation of gas-induced defects.

### 3.4. Effect of the Heat Transfer Coefficient on Porosity

**Figure 8:**
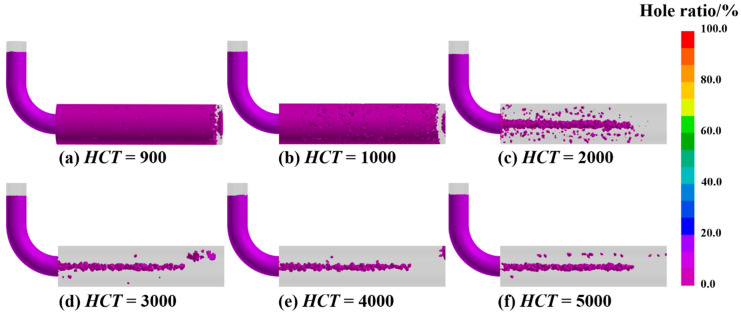
Simulated distribution of shrinkage porosity at different Interfacial HTCs, (**a**) 900, (**b**) 1000, (**c**) 2000, (**d**) 3000, (**e**) 4000, (**f**) 5000.

**Figure 9:**
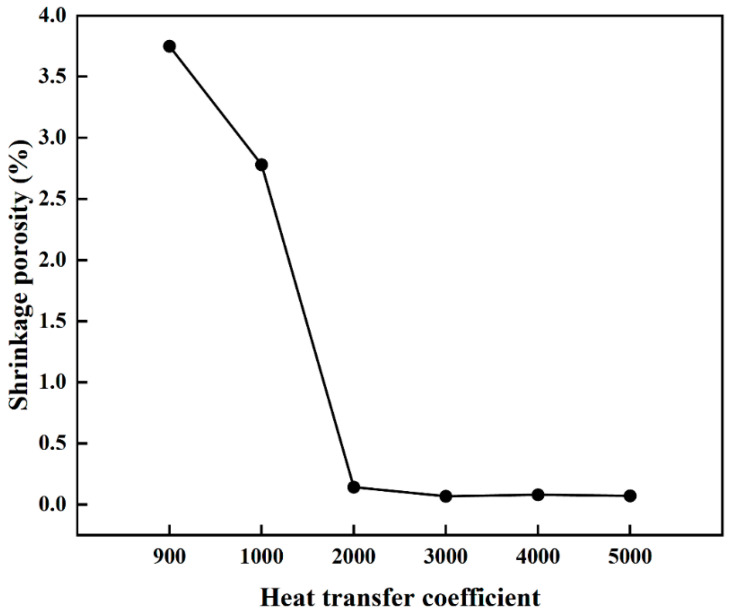
Porosity fraction as a function of the Interfacial HTC.

This study employed numerical simulation to systematically investigate the influence of varying Interfacial HTC on porosity formation during mold filling and solidification. As illustrated in Figure 8 and quantified in Figure 9, the HTC exerts a significant impact on both the morphology and total fraction of porosity under the conditions of a 330 °C PCT and a 15 s pouring time. When the HTC was below 2000 W/(m^2^·K), porosity was observed to be distributed throughout the entire ingot, which is primarily attributed to the reduced solidification rate and diminished temperature gradient that promote a broad mushy zone and obstruct interdendritic feeding paths. This behavior aligns with general findings in gravity casting, where insufficient or non-uniform cooling is known to result in molding defects due to prolonged solidification times. Conversely, when the HTC exceeded 2000 W/(m^2^·K), porosity became significantly reduced and concentrated along the central axis. This transition indicates that a higher heat transfer rate accelerates the solidification of the casting periphery and establishes a steep temperature gradient that optimizes the solidification mode to maintain unobstructed feeding channels. This mechanism is consistent with recent experimental investigations [31], which demonstrated that enhancing mold cooling efficiency (e.g., via conformal channels) significantly refines the average grain size. Such microstructural refinement, driven by rapid heat extraction, is known to improve the mechanical integrity of the casting. Furthermore, related research has highlighted that optimizing thermal conditions to control the solidification process is critical for minimizing defect susceptibility in complex alloys. Collectively, these studies support our observation that a higher HTC promotes a finer, more homogeneous structure that facilitates better feeding [32]. Statistical analysis confirms that the porosity fraction reaches a minimum at 3000 W/(m^2^·K) and subsequently plateaus, suggesting that while enhancing heat transfer effectively improves casting quality by mitigating defects associated with slow cooling, there exists a threshold beyond which further increases in HTC yield negligible benefits due to inherent thermal conductivity limitations. Therefore, 3000 W/(m^2^·K) is identified as the optimal HTC for minimizing porosity in this process.

### 3.5. Experimental Validation of Process Optimization

**Figure 10:**
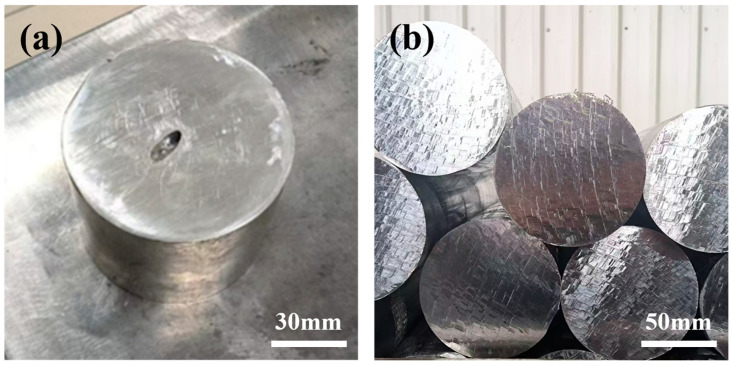
Comparison of ingot porosity before and after process improvement, (**a**) the initial process, (**b**) the improved process.

The preceding analysis identified the Interfacial HTC as the critical factor governing internal porosity formation in the ingot. The HTC itself is a function of multiple process variables, including the cooling method, casting parameters, the temperature difference between the melt and the mold, and the temperature of the cooling medium. Combining practical production experience with the numerical simulation results, we proposed and validated a strategy focused on increasing the cooling water flow rate to enhance the overall HTC during casting. Figure 10 clearly demonstrates the impact of this improvement on ingot porosity: Figure 10a shows the ingot prior to optimization of the cooling water flow rate, exhibiting significant macro-porosity defects in the central axis region. In stark contrast, Figure 10b visually confirms that the internal porosity was significantly reduced after the cooling water flow rate was increased. This result demonstrates that adjusting the cooling water flow rate to enhance heat transfer efficiency is an effective strategy for suppressing the formation of porosity in the cast ingot.

**Figure 11:**
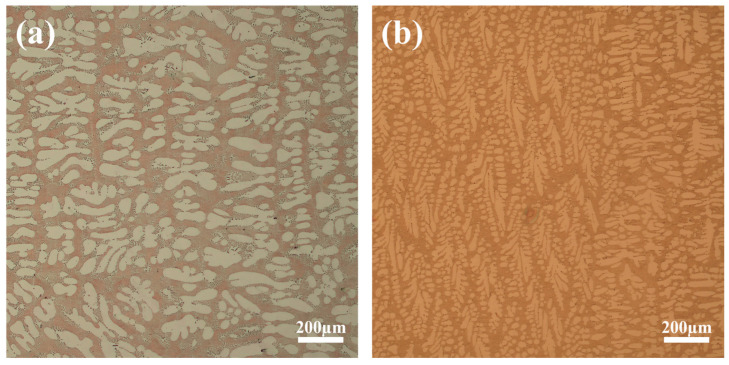
Micrographs of the ingot microstructure before and after process improvement, (**a**) Coarse dendritic structure typical of the initial process, (**b**) significantly refined dendritic structure achieved after process improvement.

Furthermore, a comparative microstructural analysis was conducted for both casting conditions, as presented in Figure 11. Prior to the process optimization, the microstructure was characterized by coarse primary dendrites; however, increasing the cooling water flow rate resulted in a discernible refinement of the dendritic structure. This morphological refinement is directly attributed to the rapid heat removal facilitated by the increased coolant flow rate, which significantly elevates the interfacial heat transfer coefficient and the subsequent solidification cooling rate. As established in solidification theory, the secondary dendrite arm spacing is inversely proportional to the cooling rate; thus, the observed refinement serves as definitive physical proof of the enhanced heat flux. As observed in experimental studies on cooling optimization, such rapid cooling regimes promote a higher nucleation density within the melt while simultaneously suppressing the excessive growth of individual dendrites [33]. Critically, this microstructural refinement provides the physical mechanism for the reduction in macroscopic porosity defects observed earlier; unlike the coarse dendritic network that easily blocks feeding channels, the refined and more homogeneous structure maintains better permeability for the residual liquid to compensate for volume contraction. Consequently, the optimized cooling condition not only eliminates the large, centerline shrinkage voids but also enhances the mechanical integrity of the casting by mitigating segregation and improving resistance to cracking. It should be noted that this study utilized a constant effective HTC and the Niyama criterion, which primarily predicts shrinkage porosity. The complex dynamics of gas evolution and the transient variation of HTC due to air gap formation were analyzed qualitatively but not coupled quantitatively in the current FEM model. Future work will focus on integrating these multi-physics phenomena for more comprehensive defect prediction.

## 4. Conclusions

This study employed the FEM to systematically elucidate how PCT and the interfacial HTC govern solidification and porosity formation in gravity-cast SAC0307 alloy ingots. The simulation revealed distinct governing mechanisms impacting porosity: PCT exerts a non-monotonic influence by primarily modulating the solidification mode affecting dispersed micro-porosity formation; specifically, an unfavorable mode at 330 °C promoted a large mushy zone and widespread feeding blockage, leading to peak porosity, whereas 350 °C achieved an optimal balance by ensuring sufficient fluidity while avoiding excessive gas absorption, consistent with Sievert’s law. Conversely, HTC plays a critical role by governing the overall solidification rate and directionality, thereby dictating defect morphology; low HTC (<2000 W/(m^2^·K)) fostered mushy solidification leading to dispersed porosity, while high HTC enforced directional solidification, isolating shrinkage defects centrally, with simulation suggesting an optimal value of 3000 W/(m^2^·K). Based on the simulation results, an optimal pouring temperature of 350 °C and an interfacial heat transfer coefficient of 3000 W/(m^2^·K) were individually determined, collectively defining the optimal process window. Importantly, the simulation’s prediction regarding the beneficial effect of increased HTC on promoting directional solidification and reducing porosity was experimentally validated. Implementing a strategy of increased cooling water flow rate successfully reduced centerline macro-porosity and promoted dendritic refinement, confirming the simulation’s capability to capture the qualitative impact of enhanced heat transfer. This work clarifies these governing mechanisms, provides a basis for defining an optimized process window based on simulation, and offers science-based guidance for improving ingot soundness.

## Figures and Tables

**Table 1:** Chemical Composition of SAC0307 Alloy (wt.%).

Cu	Ag	Pb	Ni	Others (Bi, Zn, Al)	Sn
0.7	0.3	0.05	0.05	<0.01	Balance

## Data Availability

The original contributions presented in this study are included in the article. Further inquiries can be directed to the corresponding author.
